# Analysis of the Nutritional Composition and Drought Tolerance Traits of Sweet Potato: Selection Criteria for Breeding Lines

**DOI:** 10.3390/plants11141804

**Published:** 2022-07-08

**Authors:** Sunette M. Laurie, Michael W. Bairu, Robert N. Laurie

**Affiliations:** 1 Agricultural Research Council-Vegetable, Industrial and Medicinal Plants (ARC-VIMP), Private Bag X293, Pretoria 0001, South Africa; bairum@arc.agric.za (M.W.B.); robertlaurie842@gmail.com (R.N.L.); 2 Faculty of Natural & Agricultural Sciences, School of Agricultural Sciences, Food Security and Safety Niche Area, North-West University, Private Bag X2046, Mmabatho 2735, South Africa

**Keywords:** antioxidant enzymes, drought stress, minerals, vitamin A deficiency

## Abstract

Sweet potato is an important world staple with the potential to address hunger and malnutrition. The Agricultural Research Council of South Africa has led sweet potato breeding efforts in the country since 1952 and released several important cultivars. More detailed quality assessments are necessary in addition to general breeding criteria. The present study investigated sweet potato cultivars and elite clones for (1) their nutritional composition; (2) biochemical reaction to drought stress; (3) correlate biochemical reaction to root yield for possible identification of screening methods for drought tolerance in sweet potato. Orange-fleshed cultivars, in particular Bophelo, had superior contents of Fe, Zn, Mg, Ca, Mn, and dietary fiber. Cream-fleshed cultivars, particularly Ndou, were higher in starch and carbohydrate. When sweet potato cultivars were subjected to drought stress, significant changes were noted in most antioxidant enzymes, chlorophyll and proline concentrations, and ^13^C discrimination. All of these showed significant correlations with root yield. For screening of drought tolerance, the determination of nitrate reductase, proline, and chlorophyll concentrations at 60 days after planting are recommended. Ndou was less affected by drought stress. Bophelo and Ndou, which are gaining popularity on the informal market, have superior nutritional value and are good cultivar choices for food security and addressing malnutrition.

## 1. Introduction

According to the 2021 Global Nutrition Report, 820 million people in the world suffer from hunger and over 2 billion people do not have regular access to safe, nutritious and sufficient food [1]. One in seven (20.5 million) newborns suffer from low birthweight. As an indicator of malnutrition, 149.2 million children aged < 5 years old in the world are stunted, while 45.4 million are wasted (i.e., low weight-for-height ratio). This highlights the importance of development and promotion of nutritionally balanced food crops, particularly through breeding.

Sweet potato (*Ipomoea batatas* (L.) LAM) is considered a “resilient food crop with great potential to contribute to reducing hunger in the world” [2]. The crop is the seventh most important world staple, and its production was 100 million metric tons in 2019 [3]. Sweet potatoes are cultivated in 114 countries around the world [4], and, in Africa, east African countries (i.e., Kenya, Uganda, Rwanda and Tanzania) have the highest production and shortest growing seasons [5]. In South Africa, sweet potato production has considerable commercial value [6]. In rural settings, it is considered an “indigenized” root crop because of the crop’s versatility, drought tolerance and positive role in food and nutrition security [7].

For the past seventy years, the Agricultural Research Council-Vegetable, Industrial and Medicinal Plants (ARC-VIMP) has been the lead research institution supporting the South African sweet potato industry. Formal breeding started in 1952 and aimed at providing varieties with improved root quality and yield to the local industry. At that time, farmers’ selections were grown and delivering crooked shape and defects such as veins, grooves, and cracks; therefore, there was a clear need for formal breeding. Initially, hand crosses were made among farmer’s varieties and sweet potato germplasm imported from the USA (Louisiana and South Carolina) [8]. Mafutha was the first cultivar released in 1959, followed by six more cultivars released during the 1960s (Impala, Wildebees, Griekwa, Eland, Hartebees and Koedoe) and another four until the 1980s, including Ribbok and Bosbok, which are still grown today. In 1980, the polycross system was adopted to improve crossing efficiency and seed yield. During that period, it was possible to produce 5000–10,000 or even more seeds per year [8,9]. A large number of multilocation cultivar evaluations were conducted which relates to the focus on genotype by environmental interactions reported by other researcher [10,11]. Originating from polycross progenies, Blesbok was released in 1989, a variety with wide adaptability and is still today the major local sweet potato cultivar, also produced in the SADC region and in Honduras.

New beginnings for the breeding program in 1995 placed more emphasis on informal market traits to provide improvements on Mafutha (sweet taste and drier texture with improved yield and storability). The breeding approach here was re-evaluation of clones in the germplasm collection and incorporating African imports into the polycross program. Seven cream-fleshed cultivars were released in 2003 [12]. Of these, Ndou and Monate are gaining popularity on the informal market.

The major contribution of sweet potato is through vitamin A-biofortified varieties to alleviate malnutrition. Micronutrient deficiencies remain common problems, reflecting poor dietary quality [1]. Despite shortage of data, the prevalence of vitamin A deficiency remains high in Africa and South Asia [1]. Almost half of all children in the few countries with data are affected by zinc deficiency and approximately 60% of children aged < 5 years old in low- and middle-income countries are anemic [1]. Poor diets among adults account for over 281 million years of life lost. Deaths attributable to poor diets cause more than 12 million noncommunicable disease deaths in adults. This is a quarter (26%) of all adult deaths each year [13]. Sweet potatoes are good sources of vitamins (i.e., vitamin C, folic acid, and B vitamins) and minerals (i.e., calcium, potassium, magnesium, iron, and zinc) [14,15].

During the early 2000s, developing provitamin A-enriched cultivars for nutrition security became the major objective of the ARC breeding program. Initially, the ARC promoted US varieties (e.g., Resisto and W-119) in the absence of widely adapted, sweet tasting, dry textured varieties with good yield. Moreover, conventional breeding methods were predominant using evaluation in a participatory fashion at a variety of sites with varying management practices, relying on the comprehensive germplasm collection with over 300 accessions and the support of the crop protection division with disease screening. Polycrosses consisted of US varieties and ARC-improved high dry matter material [16]. Twelve orange-fleshed varieties have been released so far from the program. Bophelo became popular shortly after its release in 2011, having a pleasant flavor, medium dry texture, and good root quality [17].

Schönfeldt, Hall, and Pretorius [18] stressed that food composition data on local and commonly consumed foods are essential and should have a larger impact on policies and program designs. Orange-fleshed sweet potato is dissociated from cream-fleshed sweet potato in terms of trans β-carotene content; consequently, orange-fleshed sweet potato is used in interventions to alleviate vitamin A deficiency. However, the two types also differ in other nutritional components and warrants detailed nutritional analysis of released cultivars and elite clones.

Drought is the primary abiotic stress affecting crop productivity and consequently food availability globally [19]. Drought tolerance breeding was reported as key in Southern Africa, with 19 drought-tolerant, orange-fleshed sweet potato cultivars released in Mozambique, the use of which has spilled over to other African countries [20]. This is of particular importance in the context of climate change. It is expected that the intensity and frequency of drought spells will increase in many parts of Africa over the next decades [20]. Drought tolerance in sweet potato is affected by several physiological processes [21,22], and considerable variation occurs among genotypes, making some susceptible to drought especially in the early stages of growth [23]. A variety of methods can be used to quantify this, e.g., measurement of chlorophyll *a* fluorescence [22], antioxidant enzyme species superoxide dismutase (SOD), glutathione reductase (GR) and ascorbate peroxidase (APX) [24], carbon isotope ^13^C [25], and proline content [26]. Screening of released cultivars and elite clones are required to investigate their biochemical reaction to identify screening methods for drought tolerance in sweet potato.

The present study investigated South African sweet potato cultivars and elite clones for (1) the range of nutrients present; (2) biochemical reactions to drought stress and to correlate biochemical reactions to root yield to possibly identify screening methods for drought tolerance in sweet potato.

## 2. Results and Discussion

### 2.1. Composite Analysis

Approximately 80–90% of the sweet potato dry matter is made up of carbohydrates, which mainly contain starch and sugars, with lesser amounts of pectins, hemicelluloses, and cellulose [14,27]. Table 1 presents a composite analysis of four orange-fleshed cultivars (i.e., Beauregard, Bophelo, Impilo, and W-119), one with yellow-orange flesh (i.e., 199062.1), and two cream-fleshed cultivars (i.e., Ndou and Monate). The ARC cream-fleshed cultivar Ndou is superior with regards to low moisture content (71.87%), high energy content (431.3 kJ 100 g^−1^), carbohydrates (22.27%), ash (0.97 g 100 g^−1^), and dietary fiber (3.37 g 100 g^−1^), except when compared to US orange-fleshed cultivar W-119.

The protein content of sweet potato was low (~2%), as in most tropical root and tuber crops, but sweet potato contained more protein than cassava and plantain [27]. Impilo and Ndou had superior protein content of 1.57% and 1.53%, respectively (Table 2). Shekhar and coworkers also reported a high percentage of carbohydrate in cream-fleshed sweet potatoes, but in their case, orange-fleshed sweet potatoes revealed higher protein [28]. Leighton [29] reported means of 1.3% for protein and 18.6% for carbohydrate content for four orange-fleshed cultivars including W-119.

### 2.2. Sugars and Starch

Sweet potato had a low glycemic index, indicating low digestibility of starch despite the high carbohydrate content [30]. Orange-fleshed cultivars, Beauregard, Bophelo, Impilo, and W-119, mostly had higher total sugar content (3.51 to 4.18 g 100g^−1^) and glucose content (1.72 to 2.557 g 100g^−1^) (Table 2). Shekhar and coworkers found higher reducing sugar content in cream-fleshed sweet potato (than in orange-fleshed sweet potatoes) [28]. Single values for starch showed a trend that Ndou, Monate, and W-119 had higher starch content. Lactose and maltose were not detected.

### 2.3. Vitamins

Orange-fleshed sweet potatoes contained very high quantities of β-carotene, the major precursor of vitamin A [15]. Based, for example, on the nutrient composition as reported in [31] and the recommended dietary allowance (RDA) for 1 to 8 year old children [32], a 100 g portion of boiled sweet potato provided substantial quantities of vitamin C (28–47% of RDA), moderate quantities of pyridoxine (12–14% of RDA), and some quantities of thiamine (8–10% of RDA) and niacin (6–8% of RDA). Both β-carotene and vitamin C function as antioxidants helping to eliminate free radicals [33].

South African cultivars Bophelo and Ndou were indicated to contain both vitamin B9 and B6 (Table 3). β-Carotene was highest in US cultivar W-119 (8293 µg 100g^−1^), followed by US Beauregard (6510 µg 100g^−1^) and ARC cultivar Bophelo (6318 µg 100g^−1^). Vitamin C was lowest in the cream-fleshed cultivars Ndou and Monate. Ojimelukwe and Okpanku found higher all-trans-β-carotene content of 8789 µg 100g^−1^ in UMUSPO 3 cultivar [34].

### 2.4. Mineral Content

Sweet potatoes are good sources of minerals [14,15]. Potassium was found to be abundant (Table 4). ARC cultivar Impilo was shown to have superior content of phosphorous (198.4 mg 100g^−1^), magnesium (136.2 mg 100g^−1^), and calcium (82.5 mg 100g^−1^). Reported mineral content in fresh sweet potato roots harvested at various location in South Africa ranged from 34 to 101 mg 100g^−1^ for calcium, 15 to 37 mg 100g^−1^ for magnesium, 28 to 58 mg 100g^−1^ for phosphorus, and 191 to 334 mg 100g^−1^ for potassium [35]. In Benin, the mineral composition ranged from 0.53 to 0.73 mg 100g^−1^ for iron, 0.23 to 0.27 mg 100g^−1^ for zinc, 23.04 to 29.97 mg 100g^−1^ for calcium, 21.30 to 25.40 mg 100g^−1^ for magnesium, 42.00 to 46.33 mg 100g^−1^ for phosphorus, 308.67 to 328.67 mg 100g^−1^ for potassium, and 29.00 to 34.00 mg 100g^−1^ for sodium [36]. Differences in mineral content are found due to soil mineral content, soil pH, concentration of exchangeable elements in addition to differences among cultivars [35].

Micro-elements zinc, manganese, iron, and copper as determined for the seven South African cultivars are presented in Table 5. Bophelo was particularly rich in manganese content (1.357 mg 100g^−1^), while iron content was generally higher in orange-fleshed cultivars (1.59 to 1.92 mg 100g^−1^) than cream-fleshed cultivars (1.28 to 1.58 mg 100g^−1^). Copper was present in higher concentrations in Beauregard and Ndou.

### 2.5. Principal Component Analysis

**Figure 1:**
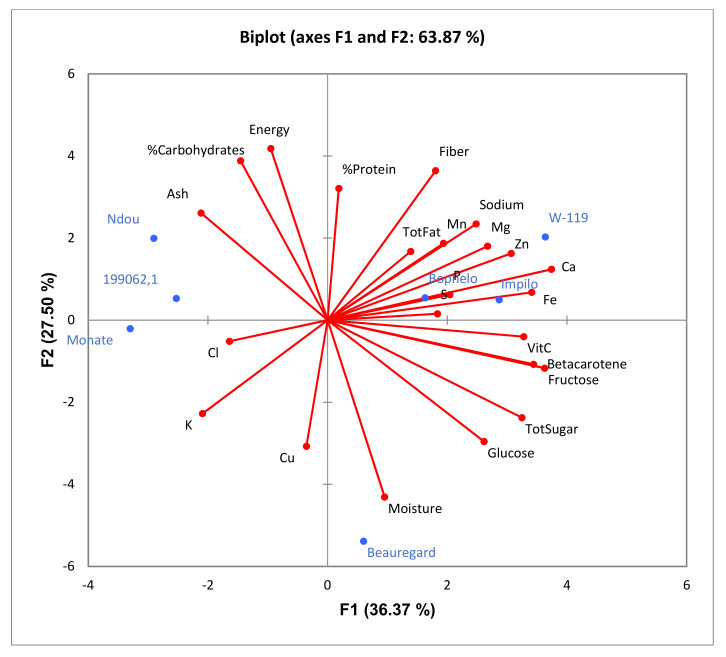
Biplot of the principal component analysis of all compositional parameters determined in seven sweet potato cultivars.

The biplot of the principal component analysis (PCA) indicated that axis 1 explained 36.3% of the variation and axis 2 27.5%; collectively 63.8% (Figure 1). Based on the factor loadings, nutrients that contributed most to variations among the cultivars were identified. Axis 1 was most associated with calcium, iron, fructose, β-carotene, vitamin C, total sugars, and zinc content. Axis 2 was most associated with moisture, energy, carbohydrates, fiber, protein, copper, and ash content. The mentioned nutrients have long vectors in the biplot. Beauregard being high in moisture content and sugars was in close proximity to these compositional components and in the opposite quadrant from energy and carbohydrate content based on the low content of those components, whereas Ndou, Monate, and 199062.1 were in close proximity of carbohydrates and high energy as well as low moisture content. Orange-fleshed varieties, W-119, Impilo, and Bophelo, were associated with β-carotene and mineral elements.

### 2.6. Correlation among Nutrients

Strong correlations were found between β-carotene and total sugar, glucose, vitamin C, calcium, manganese, and iron, while it was negatively correlated with ash and potassium content (Table 1). Protein was associated to ash, carbohydrates, energy, magnesium, and iron. Sanoussi and coworkers [36] found that iron content was significantly correlated to magnesium and calcium content while negatively correlated with zinc.

### 2.7. General Discussion on Nutritional Composition

The present study highlighted the nutritional composition of some of the different cultivars and elite clones that are important in South Africa. This is important in light of the view that determination of nutritional composition is critical in order to take “steps towards integrating nutrition education in modern agriculture crop biofortification programs more effectively” [37].

As highlighted by Mwanga and coworkers [2], breeding for orange-fleshed cultivars with high iron and zinc content has been a focus of sweet potato breeding in subSaharan Africa. Biofortification of sweet potato with Fe and Zn could help in ameliorating the negative effects of these micronutrient deficiencies. The breeding efforts in Mozambique resulted in the release of MUSG15052-2, an orange-fleshed cultivar with high Fe levels of 4.4 mg/100 g, DW. This cultivar was used in Fe bioavailability studies in Malawi in 2019 [38].

It was found that orange-fleshed cultivars, had superior contents of Fe, Zn, Mg, Ca, and Mn as well as dietary fiber. Cream-fleshed cultivars were higher in starch and carbohydrate. Shekhar and coworkers reported a high percentage of carbohydrate, reducing sugar and phenolics in white-fleshed sweet potatoes, while orange-fleshed sweet potatoes revealed higher protein, flavonoids, anthocyanins, and carotenoids [28]. As a follow-up to the present study, Phahlane and coworkers [39] characterized the phenolic compounds of five of the seven cultivars investigated here.

### 2.8. Results of Biochemical Assays Screening for Drought Tolerance

#### 2.8.1. Ascorbate Peroxidase (APX)

**Figure 2:**
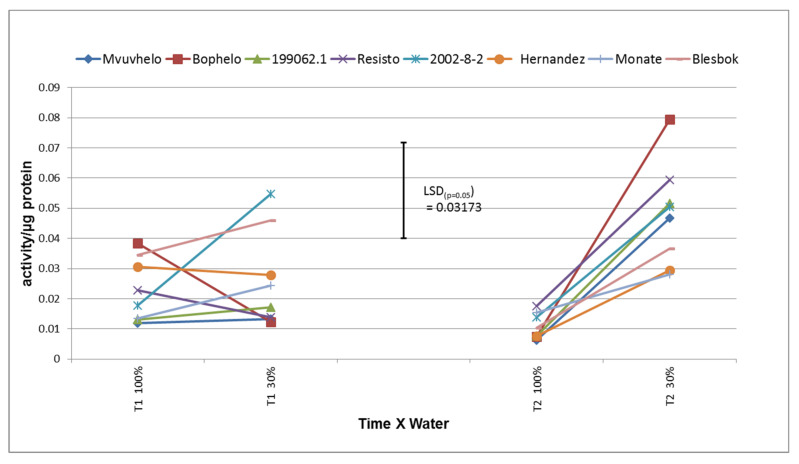
Ascorbate peroxidase activity (APX) of eight sweet potato cultivars in reaction to drought. LSD_(***p*** = 0.05)_ = 0.03173; T1 = 60 days after planting, T2 = 120 days after planting, 100% = control treatment, 30% = severe stress treatment.

A strong reaction was found in sweet potato to drought. Drought caused significant increases in APX activity in most of the leaves especially during time 2 (T2), which was determined 120 days after planting (Figure 2). This correlates with the findings of Zhang and coworkers [40] which observed increases in APX, a defensive response, over the growth cycle of sweet potato plants subjected to drought stress. Ascorbate peroxidase ranged from 0.01 activity/µg protein in control conditions to 0.09 activity/µg protein in drought-stressed conditions. Sweet potato elite line 2002-8-2 showed a significant increase in APX activity from the control to severe stress conditions, giving rise to the possible increase in H_2_O_2_ levels during the drought condition which then could be neutralized by APX action. Cultivars Mvuvhelo, 199062.1, Monate, and Blesbok also showed increases in APX activity, but these proved insignificant, indicating that the stress was affecting the antioxidant system, specifically the APX enzyme system, to a very low degree. This was also indicated in [41], where non-significant increases in APX activity were observed when wheat plants were subjected to drought stress. The sweet potato plants in the control treatment of T1 (60 days after planting) did not show any significant difference to the plants in the control treatment at T2. Lu and coworkers [42] showed that expression of APX in chloroplasts of sweet potato enhanced drought resistance and capacity for recovery from drought stress.

At T2, the majority of the cultivars experienced an increase in APX activity. This gives rise to the probability that the drought stress resulted in the formation of reactive oxidant species that forces the plants to react by elevating the activity of APX [43]. The cultivar Bophelo showed the most intense reaction to the stress at T2 with regard to APX activity, showing a significant difference (increase) compared to Hernandez, Monate, and Blesbok. Bophelo did not show a significant response to the drought stress at T1, but the severity of the stress forced the plants to react by increasing the levels of APX activity at T2. The small nonsignificant increase in APX activity from T1 to T2 in the severe stress treatment for Monate and Hernandez could be the result of very little response from the peroxidase antioxidant system of the plants to ensure the decomposition of H_2_O_2_. No explanation can be rendered for the relative low average APX values in the severe stress treatment compared to the average APX values in the control treatment at 120 DAP other than the fact that the sweet potato plants did not react intensely to the stress to detectable increased levels of APX.

Although the majority of sweet potato cultivars and lines exhibited moderate elevated levels of APX during the drought stress, it appears that peroxidase is not one of the antioxidant pathways sweet potato plants use to decrease the reactive oxygen species levels during drought stress periods, therefore not an efficient selection criterion for drought tolerance in sweet potatoes.

#### 2.8.2. Super Oxide Dismutase (SOD)

**Figure 3:**
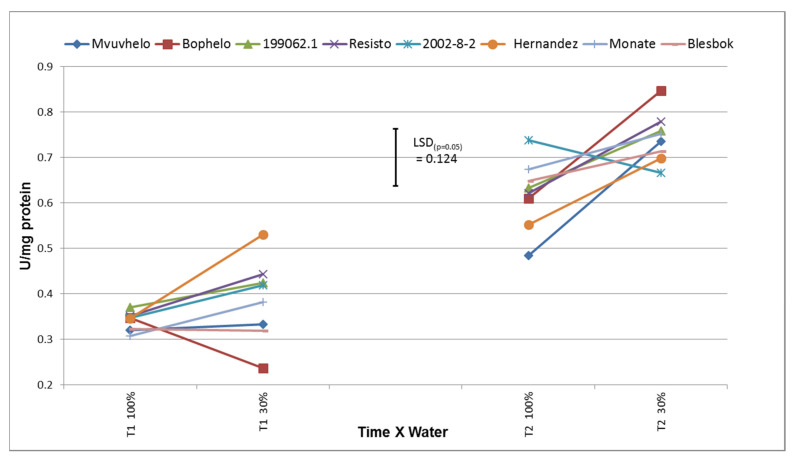
Activity levels of the superoxide dismutase (SOD) enzyme in the leaves of eight sweet potato cultivars and lines subjected to control and drought stress conditions. LSD_(***p*** = 0.05)_ = 0.124; T1 = 60 days after planting; T2 = 120 days after planting; 100% = control treatment; 30% = severe stress treatment.

An increase in SOD activity was observed in sweet potato cultivars subjected to drought stress. SOD activities varied from 0.350 U/mg protein in control conditions to 0.85 U/mg protein in drought stress conditions (Figure 3). This is similar to the findings in [40,44] that showed increases in SOD activity in soybean cultivars subjected to drought stress. Significant differences between the cultivars were observed at T1 and T2 for the severe stress treatment (Figure 3). The values of SOD activity at severe stress at T2 in general were also higher and could possibly be ascribed to more drought experienced by the cultivars and the resulting increase in O_2_ radical levels in the leaves. During T1, the cultivar Hernandez showed a significant increase in SOD activity in severe stress conditions that was possibly the result of increased accumulation of reactive oxygen species to be reflective of an increased stress condition [45]. The cultivar Bophelo showed a non-significant decline in SOD activity at T1 from the control to the severe stress treatment, which could be the result of either water contamination or error in leaf identification.

#### 2.8.3. Glutathione Reductase (GR)

**Figure 4:**
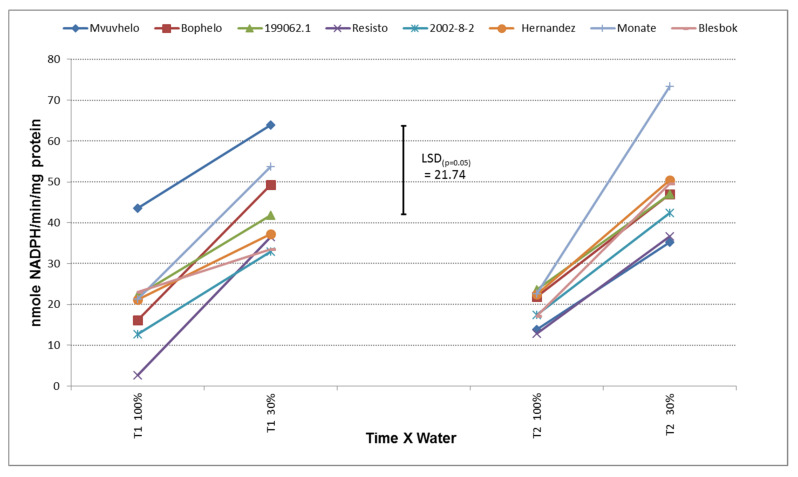
Glutathione reductase (GR) values in the leaves of eight sweet potato cultivars subjected to drought. LSD_(***p*** = 0.05)_ = 21.74; T1 = 60 days after planting; T2 = 120 days after planting. 100% = control treatment; 30% = severe stress treatment.

The GR concentrations in the cultivars subjected to drought stress showed an increase from the control to the stress treatment at both T1 to T2 (Figure 4). Although the GR increase between the control and severe stress treatment was to the same extent for each of the cultivars, the simultaneous increase in SOD activity confirms the possibility of drought tolerance [46]. GR activities indicated significant differences between breeding clones as a result of the drought stress. GR activities ranged from 2 nmoles NADPH/min/mg protein in control conditions to 73 nmoles NADPH/min/mg protein in stressed conditions (Figure 4). Significant differences in GR activity between the cultivars were also detected in the control treatment of T1. This could possibly indicate the genotypic diversity between the cultivars for GR activity at control conditions. At T1 the cultivar Mvuvhelo showed an increase in GR activity, although this was not significantly different from the value in the control treatment.

The cultivar Monate did show a significant increase in GR from the control to the severe stress treatment at T2. The insignificant difference between values in severe stress conditions at T1 and T2 could indicate that the plants had reached their maximum response to the stress at T2 and that a possible degradation of the GR protein occurred. Masoumi and coworkers [44] observed a decline in GR activity in drought experiments which could indicate tissue degradation that leads to a decline in GR values.

#### 2.8.4. Nitrate Reductase (NR)

**Figure 5:**
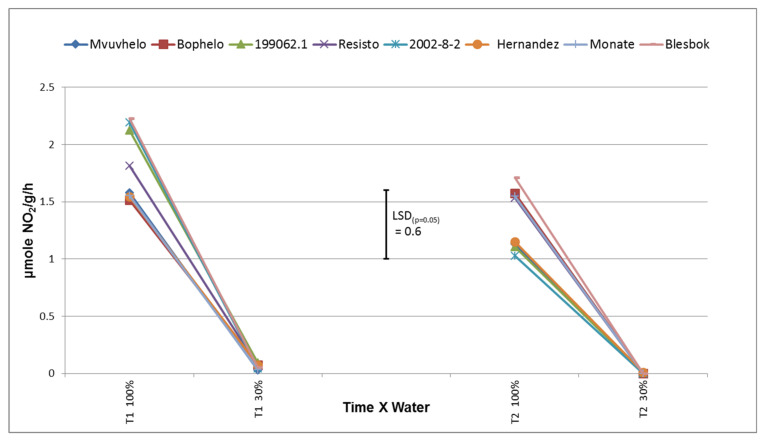
Activity of nitrate reductase (NR) enzyme displayed through the amount of NO_2_ consumed in eight sweet potato cultivars as a result of drought stress. LSD_(***p*** = 0.05)_ = 0.6; T1 = 60 days after planting; T2 = 120 days after planting. 100% = control treatment, 30% = severe stress treatment.

NR activity was severely impeded by the drought stress conditions (Figure 5). Mazid and coworkers [47] reported that NR plays a role in regulation of NO_3_ assimilation and N-fixation under photosynthetic modulation. The effect of the drought was to such a degree that no significant difference could be detected among genotypes. It is assumed that a severe decline in enzyme activity in these trials will have a negative effect on the growth of the plants. A decline in photosynthesis also has a negative effect on nitrate reductase [48], which might fit into our reasoning that stomatal conductance will interfere with the photosynthesis rate and, hence, cause a decline in NR levels. NR ranged from 2.4 µmole NO_2_/g/h in control conditions to 0.0016 µmole NO_2_/g/h in drought stress conditions (results not shown).

Drought had a severe negative effect on NR activities in sweet potato leaves (Figure 5). NR is a key enzyme in the nitrogen assimilation pathway, which is actively controlled during drought [24]. A significant decline in NR activity was observed at severe stress at T1. Significant genotypic differences were displayed in the control treatment at both T1 and T2. The activity of NR at T2 in the control treatment was significantly lower than the control treatment at T1 for some of the cultivars.

No significant differences could be detected between the cultivars in NR activity under severe stress conditions at either T1 or T2, at which time the activities were already very low, possibly due to the fact of protein degradation [24]. This reduction in NR will have an effect on the growth of the plant as nitrogen is an important component in the formation of numerous substances in the plant.

#### 2.8.5. Chlorophyll Content Index (CCI)

**Figure 6:**
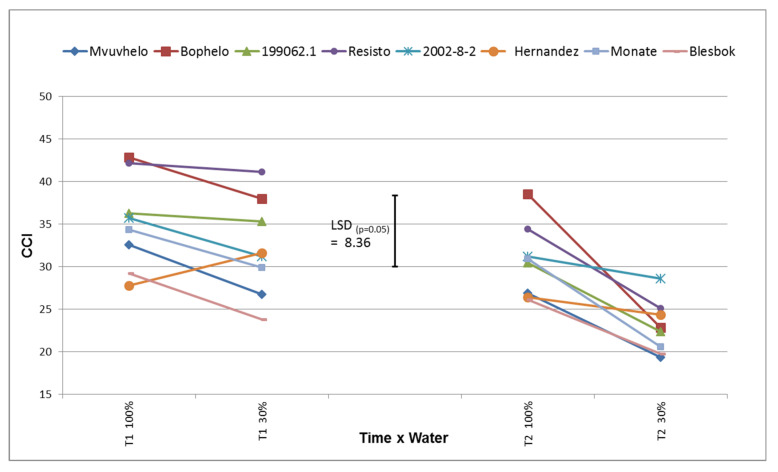
Chlorophyll content index (CCI) of eight sweet potato cultivars subjected to drought stress. LSD_(***p*** = 0.05)_ = 8.36; T1 = 60 days after planting; T2 = 120 days after planting. 100% = control treatment; 30% = severe stress treatment.

The CCI of the plants subjected to drought stress at T1 did not decline significantly from the control to the severe stress treatment (Figure 6), though there was a declining trend. Zhang and coworkers [40] detected a reduction in CCI at 40, 60, 80, and 100 DAP during drought stress. The cultivar Hernandez exhibited an increase in CCI, although not significant, from control to severe stress conditions. The cultivars displayed various levels of CCI in the control treatment, which extended to the severe stress treatment at T1. This could be ascribed to the genetic difference between the cultivars, which will affect the photosynthetic capacity of the different cultivars.

A significant and more powerful decline in CCI values was observed at T2 in all the stressed plants. Nikolaeva and coworkers [49] found a significant decline in CCI when subjecting wheat plants to drought stress. Monate, Resisto, and Bophelo showed significant reduction from the control to the stress treatment. It is speculated that this could possibly have an effect on the photosynthetic systems of the plant and may have caused the reduction in growth seen in the canopy and stem development. The breakdown of chlorophyll may also have an influence on the intensity of the antioxidant enzyme system, hence, the relative low values recorded. Heider and coworkers [50] considered CCI as a potential marker for breeding for heat tolerance.

#### 2.8.6. ^13^C Discrimination (Δ^13^C)

**Figure 7:**
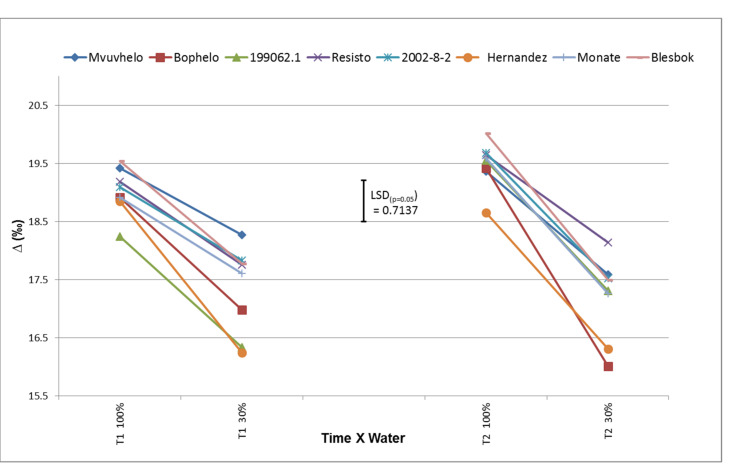
^13^C isotope discrimination (∆^13^C) values in leaves of eight sweet potato cultivars subjected to drought and control conditions in Trial 3. LSD_(***p*** = 0.05)_ = 0.7137; T1 = 60 days after planting; T2 = 120 days after planting. 100% = control treatment; 30% = severe stress treatment. Each value is the mean of 3 measurements with 3 repeats per cultivar.

Significant differences in carbon discrimination values were observed for the water x variety combination. Changes in Δ^13^C are displayed in Figure 7. A decrease in Δ^13^C values was observed for all the cultivars, from the control treatment to the severe stress treatment, at both T1 and T2. These findings were confirmed in [25,51,52], which also observed declines in Δ^13^C in potato, wheat, and cotton plants, respectively, subjected to drought. In addition, Gouveia and coworkers [53] noted a decrease in Δ^13^C in sweet potato subjected to drought stress based on NIRS estimation. Significant differences between the Δ^13^C in the control treatment and the severe stress treatment at T1 were observed, with cultivars 199062.1 and Hernandez having the lowest values. The cultivar Hernandez continued to display a low isotope discrimination value at T2 while 199062.1 had an increase in Δ^13^C value. The lower Δ^13^C value for Hernandez indicated that the cultivar assimilated more ^13^C at T2 as well, while more discrimination occurred in 199062.1 as the stress increased. This might lead to a possible decline in biomass [52] as the stress increases. No significant difference in carbon isotope discrimination could be observed between the cultivars Mvuvhelo, Resisto, Blesbok, and Monate in the severe stress treatment for both times, which could mean that these cultivars cannot be ranked genotypically based on carbon assimilation alone during drought stress.

#### 2.8.7. Free Proline Content

**Figure 8:**
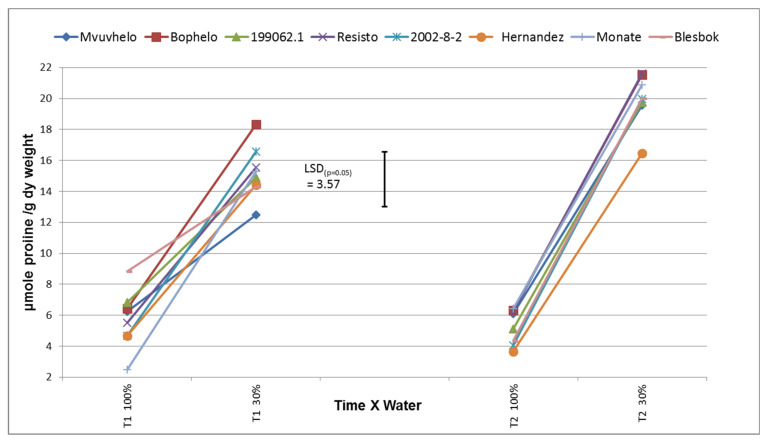
Proline concentrations in the leaves of eight sweet potato cultivars and breeding lines subjected to drought. LSD_(***p*** = 0.05)_ = 3.57; T1 = 60 days after planting; T2 = 120 days after planting. 100% = control treatment; 30% = severe stress treatment.

It was possible to distinguish and obtain significant differences among genotypes for free proline content (Figure 8). Proline content ranged from 2 µmole/g in control conditions to 22 µmole/g in drought stress conditions. Significant increases in free proline concentrations from the control treatments to the severe stress treatments were observed. Significant increases in general, up to five-fold between the control and the severe stress treatments, were observed in T2 compared to the 2.5-fold increase in T1. This indicates that the plants experienced a drought condition that led to an increase in proline production either via the transport of free proline from the root system [54], increased enzymatic production [55], or the breakdown of protein. In the severe stress treatment at T1, the cultivar Bophelo produced the highest level of free proline, and Mvuvhelo the lowest.

#### 2.8.8. Sweet Potato Yield

The results of root yield and water use efficiency were discussed in detail in [56]. In summary, drought stress had a significant effect on the yield of the genotypes. Yield was reduced, on average, by 85% at stress compared to control treatment. This correlated with the findings by van Heerden and Laurie [57] which also showed significant differences between two sweet potato genotypes subjected to drought stress. Lewthwaite and Triggs [58] indicated that the influence of drought on yield components differed with specific clones, both in the number of storage roots formed (*p* < 0.001) and average root weight (*p* < 0.001). Meanwhile, Kivuvu [59] concluded that mechanisms for drought mitigation include lower numbers and sizes of storage roots, proliferation of pencil fibrous roots, reducing vine branching, elongation of fibrous roots, increased shooting, mature leaf pubescence, and ability to retain CCI for the sweet potato clones evaluated. Solis and coworkers reported from greenhouse drought for 5 and 10 DAT reduced the number of storage roots by 42% and 66%, respectively, compared with the controls [60]. Field drought resulted in a 49% reduction in US #1 storage root yield compared with the irrigated condition.

#### 2.8.9. Relationship between Biochemical Compounds and Yield

Significant correlations were found between yield and all biochemical variables (Table 6). Yield and GR, APX, SOD, and proline had a moderate to strong negative correlations, while CCI and NR had strong positive correlations with yield.

The present study provided a comprehensive investigation and comparison of a large number of variables related to the reaction of the sweet potato plant to drought. It could be seen from the present results that antioxidant enzymes are possibly responsible for the positive reaction during drought. An effective method for screening genotypes for drought tolerance after a short growing period will be useful to breeders to knockout lines and, thus, reduce the cost of screening. Moderate to strong positive correlations were found between yield and NR activity, CCI and discrimination against ^13^C, as well as strong negative relationships with the proline content, and moderate to strong negative relationship of yield with antioxidant enzymes ascorbate peroxidase, glutathione reductase, and superoxide dismutase. The mentioned variables can thus be considered as screening tools for the identification of drought-tolerant genotypes.

Although positive results have been shown in the literature, where discrimination against ^13^C has been used to identify drought tolerant genotypes in other crops, there are several contradictions regarding the procedure that may cause doubt on this type of screening. In addition, the analysis of ^13^C is time consuming and expensive, contributing to the argument that it might not bring about a cost-saving and quick method for drought screening. Likewise, the determination of APX, SOD, and GR is quite tedious. Proline concentration has been used with success in a number of crops. Determination of the biochemical variables can therefore be narrowed down to NR, proline content, and extracted chlorophyll concentrations—all of which are relatively easy to quantify. We further recommend that such assays can be conducted at 60 DAP.

## 3. Conclusions

The nutritional composition was determined for some important sweet potato cultivars and elite clones which are grown in South Africa or are in advanced stage of development. This is important for recommending cultivars to address malnutrition and food security. It was shown that orange-fleshed sweet potato are superior in Fe, Zn, Mg, Ca, and Mn concentrations and dietary fiber, while cream-fleshed cultivars generally contain higher concentrations of starch and carbohydrate. The comprehensive investigation of variables associated with the sweet potato plant’s reaction to drought indicated antioxidant enzymes which are possibly responsible for the positive reaction during drought. It is recommended that the determination of NR, proline and extracted chlorophyll concentrations at 60 DAP are effective methods for screening genotypes for drought tolerance

Drought stress represents a global constraint for sweet potato production because most of the sweet potato production occurs in semiarid regions. Considering the complexity of the physiological and genetic mechanisms associated with stress tolerance, more emphasis on a genomics-based understanding of the stress response of sweet potato will help develop strategies to sustain productivity in stressful environments.

The presented results provide broader characterization of sweet potato cultivars and elite clones from South Africa and contribute to recommendations for production and is an important step of chemotyping for further genome-wide association studies.

## 4. Materials and Methods

### 4.1. Breeding Procedures

**Figure 9:**
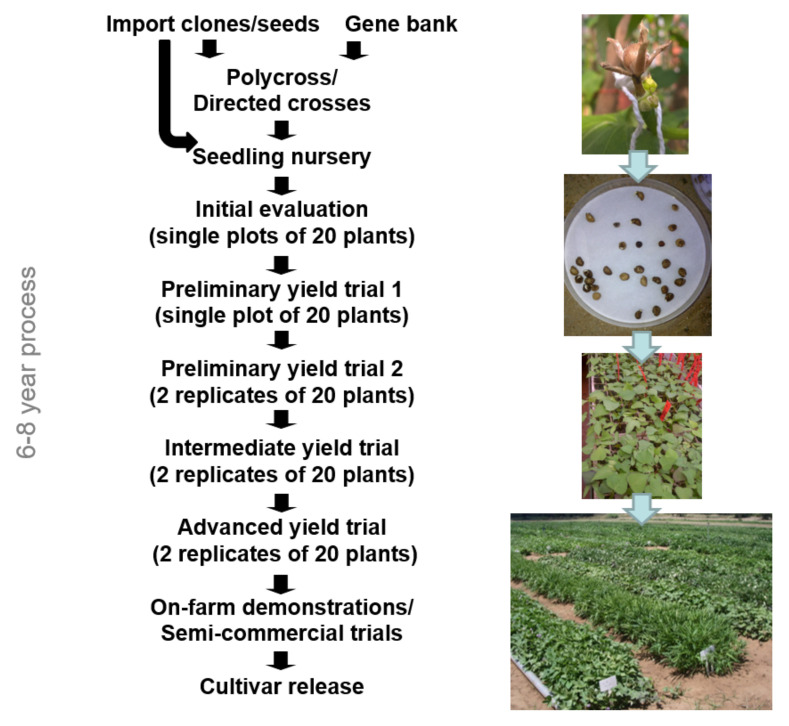
Schematic representation of the breeding cycle.

The breeding scheme is presented in Figure 9. Desired characteristics were developed in the sweet potato breeding population through the polycross method or seed imports from breeding programs of the International Potato Centre in Mozambique and Uganda. To raise seedlings from hybridization or seed imports, seeds were scarified with concentrated sulfuric acid for 30 min, floated in water to remove nonviable seeds, placed in Petri dishes to germinate, and then sown in seedling trays. After one month, the seedlings were transplanted to the field seedling nursery (2000–4000 lines). At harvesting, two to four percent were selected for further evaluation. Selection was based on yield (single plant basis), flesh color, absence of root defects, and raw taste.

Approximately 50–80 lines were evaluated in the initial evaluation and preliminary yield trial 1 in single plots. Evaluations in the preliminary yield trial 2 and intermediate yield trial consisted of two replicates of 20 plants, while the advanced yield trial was planted with three replicates of 20 plants. Data collection included: color, % per root shape, skin damage, % and severity of defects, oxidation rating, dry matter content, keeping ability over 21 days at room temperature, number and kg per marketable and unmarketable classes, percentage per marketable size class, and total yield. Sweet potatoes of each line were cooked and evaluated by a small panel consisting of ARC personnel. Additionally, total carotenoid content of intermediate and advanced lines were determined with a spectrophotometer. In each phase, the number of clones reduced as clones with undesirable traits were discarded. This process normally took 6–8 years before release of cultivars. However, a genotype may be “fast-tracked” by skipping an evaluation phase, at the discretion of the breeder. 

### 4.2. Plant Material and Sampling for Nutritional Analysis

Six sweet potato cultivars with flesh color varying from cream to dark orange were grown in a randomized complete block design at the Agricultural Research Council-Vegetable, Industrial and Medicinal Plants (ARC-VIMP), Roodeplaat, Pretoria (25.604° S, 28.345° E; 1189 m altitude), during the 2012/13 growing season. The cultivars represented popular cultivars promoted in South Africa to address vitamin A deficiency and food security [16]. The soil type was sandy–loam (15% clay in upper 600 mm), and the pH range was 6.5–7.0. For fertilizer application, based on a soil analysis, 500 kg ha^−^^1^ chemical fertilizer mix N:P:K (18.5% N, 0% P, 18.5% K) was applied before planting, followed by 150 kg ha^−^^1^ ammonium sulphate (21% N) at 15 and 30 days after planting, and 200 kg ha^−^^1^ potassium sulphate (40% K) at 21 and 42 days after planting. The total fertilizer application therefore was 156, 50, and 252 kg ha^−^^1^ N, P, and K, respectively. At five months after planting, optimal for the climatic area, the sweet potatoes were harvested. Medium–large storage roots (400–600 g) of each cultivar were randomly sampled over the three replicates of each cultivar for determination of nutrient content.

### 4.3. Nutritional Analysis

The roots were cut longitudinally, and two opposite quarters homogenized in a food processor. The homogenized samples were divided into two. One part was cooled to 7 °C and subsamples transported to Microchem Specialized Lab Services in Cape Town for composite analysis and individual sugars and starch analysis (Table 7), and to Cape Town Peninsula University for analysis of vitamin C and β-carotene content. The other part was freeze-dried, and 1 g of each sample sent to the James Hutton Institute in United Kingdom for mineral determination.

Analyses of β-carotene were carried out according to a validated method developed for orange-fleshed sweet potato [62,63]. This involved the purification of a β-carotene standard (i.e., synthetic, crystalline, Type II, and product C-4582) from Sigma Chemical Co., and quantitative analysis by high-performance liquid chromatography (HPLC). The mobile phase consisted of acetonitrile (containing 0.05% triethylamine):methanol:ethyl acetate (80:10:10), with a flow rate of 0.7 mL min^−1^. The detector wavelength was set at 450 nm for the detection of β-carotene.

Total vitamin C (ascorbic acid + dehydro-ascorbic acid) was determined using the HPLC-UV method as described by [64,65]. Vitamin C was analyzed using a Thermo Finnigan Spectra System HPLC with the UV wavelength detector set at 245 nm. The flow rate of the mobile phase (0.01% solution of sulfuric acid, pH 2.6) was set at 0.9 mL/min. Quantification of the vitamin C in the samples was performed by setting up a standard curve using L-ascorbic acid with concentrations varying from 5 to 50 μg mL^−1^.

Mineral analysis included K, Cl, P, Na, Mg, Ca, Cu, Fe, Zn, and Mn. Each powdered sample (2 g) was subjected to measurement through the inductively coupled plasma optical emission spectrometric (ICP-OES) determination according to [66].

### 4.4. Experimental Conditions for Drought Stress Screening

A drought stress trial was executed at the ARC-VIMP, Roodeplaat, Pretoria (25.604° S, 28.345° E; 1189 m altitude). Eight sweet potato cultivars were planted in a split plot design with three replicates (6 rows of 3 plants per plot) in a rainout shelter with a plant spacing 0.8 by 0.3 m. Water management was conducted by monitoring the soil water content with a capacitance probe (Ventek CC, Pretoria, South Africa). The 100% irrigation treatment (open field) received the full complement of plant available water through a sprinkler irrigation system once soil water had depleted to 70%, while the stress treatment (rainout shelter) received 30% of the calculated water that the 100% treatment received. This resulted in continuous drying out of the soil in the 30% treatments. Based on soil analysis, preplant fertilizer applied involved 500 kg ha^−1^ 1:0:1 N:P:K fertilizer mix (37% active compounds), and top dressing of 130 kg ha^−1^ ammonium sulphate at 14 and 30 days after planting and 200 kg ha^−1^ K_2_SO_4_ at 20 and 40 days after planting (DAP).

### 4.5. Biochemical Assays Related to Drought Stress

Sampling of sweet potato leaves took place twice during the trial period at 60 DAP (T1) and 120 DAP (T2) before sunrise. Thirty leaves, the fifth from the apical tip, were harvested from each cultivar for each repeat for each of the treatments, stored immediately at −80 °C and subsequently freeze-dried.

#### 4.5.1. Ascorbate Peroxidase (APX)

The activity of APX was determined following the method in [41]. Phosphate buffer (50 mM) and ascorbic acid (0.25 mM) were mixed with the enzyme extract. The reaction was started by adding 1 mM H_2_O_2_ and following the reduction for 1 min measured at 265 nm with a Beckman Coulter DU 800 UV/Vis spectrophotometer (Beckman Coulter Inc., Brea, CA, USA).

#### 4.5.2. Superoxide Dismutase (SOD)

The activity determination was executed using the method in [67]. The results were based on the formation of nitrite from hydroxylammonium chloride in the presence of SOD. A solution was prepared containing 65 mM potassium phosphate buffer, xanthine reagents, and hydroxyl ammonium chloride. After the addition of the enzyme extract the mixture was incubated at 25 °C for 20 min. The addition of sulphanilic acid and α-naphtylamine and subsequent 20 min incubation period allowed for color formation that was determined spectrophotometrically at 530 nm with a Multiscan EX multiplate reader (MTX Lab Systems, McLean, VA, USA).

#### 4.5.3. Glutathione Reductase (GR)

The GR activity was determined using the method in [68]. The enzyme was extracted where after an aliquot of the extraction mixture was added to a mixture of oxidized glutathione (GSSG) (0.25 mM), Tris (50 mM), and EDTA (0.5 mM). NADPH (0.125 mM) was added to the solution and the oxidation of NADPH followed spectrophotometrically at 340 nm over a period of 1 min. Enzyme activities were expressed as enzyme units (U) g^−1^ dry weight.

#### 4.5.4. Nitrate Reductase (NR)

Each sample (0.04 g) was extracted according to the method in [69]. Each leaf tissue sample, in triplicate, was ground in liquid nitrogen. Extraction was performed in buffer containing 50 mM MOPS-NaOH pH 7.5, 10 mM MgCl_2_, 1 mM EDTA, 5 mM dithiothreitol, and 0.1% (v v^−1^) Triton X-100. The extracts were centrifuged at 20,000× *g* for 2 min and the supernatants used immediately for the assays in a reaction mixture containing 1 mL of 0.1 M potassium phosphate buffer, 0.2 mL 0.1 M KNO_3_, 0.5 mL 1.36 mM NADPH, 0.2 mL enzyme extract. Reaction mixtures were incubated at 27 °C for 15 min and the reaction stopped by the addition of 1 mL 1% w v^−1^ sulfanilamide in 1.5 M HCl. N-(1 napthyl) ethylene diamine hydrochloride reagent was added (1 mL of 0.02 % (w v^−1^)) and the contents mixed by inverting the tubes. The absorbency was determined by reading each sample against its own blank (complete except for NADPH) in a Beckman DU 800 spectrophotometer at 540 nm and the activity calculated as µmole NO_2_ g^−1^ h^−1^.

#### 4.5.5. Chlorophyll Content Index (CCI)

CCI measurements were performed by using a CCM 200 chlorophyll reader (Opti-Sciences). The standard protocol, as indicated by the manufacturer’s guide, for calibration and readings were conducted during the two measurement periods, namely, 60 and 120 days after planting (DAP) (T1 and T2, respectively). The 5th leaf from the apical tip was read from 3 stems of randomly selected plants per repeat.

#### 4.5.6. Carbon Isotope Discrimination

Carbon isotope assays were conducted at the Department of Archeology, University of Cape Town, and were carried out on a Thermo Delta V stable light isotope ratio mass spectrometer interfaced via a Conflo IV with a Thermo Flash 2000 elemental analyzer. Working standards were combusted regularly, and the results of these analyses were used to normalize the sample results against international standards. The results are reported relative to the standards, VPDB for carbon and air for nitrogen. The ratio of carbon isotopes were determined as δ^13^C calculated relative to the Vienna Chicago PDB (Pee Dee Belemnite) marine limestone standard. Discrimination values were calculated using the respective carbon isotope ratio values as supplied by the University of Cape Town. Carbon isotope discrimination (∆^13^C) was calculated as:

∆^13^C (‰) = (δ_air_ − δ_plant_)/(1 + δ_plant_/1000)

where δ_air_ is the carbon isotope ratio of the air ~−8‰, and δ_plant_ is the carbon isotope ratio of the leaf sample.

#### 4.5.7. Proline Analysis

The free proline content was determined following the procedure described in [70].

#### 4.5.8. Yield Parameters

The total aboveground biomass and the storage root yield were determined at the end of each season (five months) as reported in [56].

#### 4.5.9. Statistical Analysis

Data obtained for the various measurements were subjected to ANOVA using GenStat 64 bit, release 18.2 (PC/Windows 8) (VSN International, Hemel Hempstead, UK). XLSTAT was employed for multivariate analyses, Pearson correlation among nutrients and minerals, agglomerative hierarchical clustering, and principal component analysis to investigate which nutrients contribute most to variation.

All biochemical measurements were subjected to an ANOVA. Means of significant effects were separated using Fishers’ *t*-LSD (least significant difference) at a 5% level of significance. Statistical analyses were conducted using GenStat for Windows 15th Edition (VSN International, Hemel Hempstead, UK). Pearson correlation approach was used to establish the possible linear relationship between enzyme concentration and yield determined by [56].

## Figures and Tables

**Table 1:** Composite analysis (fresh weight basis) of sweet potato cultivars with varying flesh colors.

Cultivar	Moisture	Energy	Protein	Total Fat	Ash	Carbohydrates	Dietary Fiber	Starch *
	(g 100 g^−1^)	(kJ 100 g^−1^)	(%)	(g 100 g^−1^)	(g 100 g^−1^)	(%)	(g 100 g^−1^)	(g 100 g^−1^)
Beauregard	82.40 a	266.8 d	0.80 d	ND	0.70 d	13.80 e	2.37 c	9.57
Bophelo	76.10 bc	355.2 c	1.00 c	ND	0.87 c	17.97 d	4.07 a	12.87
Impilo	77.07 b	344.2 c	1.57 a	0.10 ab	0.90 bc	16.67 d	3.73 ab	11.37
W-119	72.47 de	430.3 a	1.20 b	0.10 ab	0.70 d	21.57 ab	3.93 a	16.77
199062.1	74.30 cd	391.7 b	1.10 bc	0.17 a	0.97 ab	19.97 c	3.57 ab	15.67
Monate	74.77 c	389.2 b	1.07 bc	ND	0.80 c	20.47 bc	2.87 bc	16.33
Ndou	71.87 e	431.3 a	1.53 a	ND	0.97 a	22.27 a	3.37 abc	16.43
Mean	75.57	372.6	1.180	0.058	0.842	18.95	3.41	14.14
*p*-Value	<0.001	<0.001	<0.001	<0.001	<0.001	<0.001	<0.001	-
SEM	0.60	9.31	0.069	0.007	0.026	0.512	0.339	-
LSD	1.809	28.23	0.209	0.022	0.077	1.552	1.029	-
CV%	1.4	4.3	10.1	21.4	5.3	4.7	17.2	-

Values in a column followed by the same letter are not significantly different (*p* = 0.05). * Results from single-value determinations.

**Table 2:** Total sugar, fructose, and glucose content of sweet potato cultivars with varying flesh colors.

Cultivar	Total Sugar	Fructose	Glucose
	(g 100g^−1^)	(g 100g^−1^)	(g 100g^−1^)
Beauregard	4.183 a	1.497	2.557 a
Bophelo	3.517 abc	1.433	1.730 bc
Impilo	3.657 ab	1.650	2.010 ab
W-119	4.020 a	1.523	2.273 ab
199062.1	2.970 bc	1.290	1.683 bc
Monate	3.100 bc	1.210	1.693 bc
Ndou	2.803 c	1.100	1.293 c
Mean	3.464	1.386	1.891
*p*-Value	0.018	0.638	0.008
SEM	0.270	0.228	0.195
LSD	0.820	NS	0.591
CV%	13.50	28.4	17.8

Values in a column followed by the same letter are not significantly different (*p* = 0.05).

**Table 3:** Vitamins B9, B6, and C and trans β-carotene content of sweet potato cultivars with varying flesh colors.

Cultivar	Vitamin B9	Vitamin B6	β-Carotene	Vitamin C
	mg 100g^−1^	mg 100g^−1^	µg 100g^−1^	mg 100g^−1^
Beauregard	0.00	0.000 c	6510 b	5.42 ab
Bophelo	0.07	0.410 a	6318 b	5.58 a
Impilo	0.00	0.000 c	3837 c	4.82 ab
W-119	0.00	0.000 c	8293 a	7.02 a
199062.1	0.00	0.000 c	2561 d	4.94 ab
Monate	0.00	0.000 c	0 e	1.45 c
Ndou	0.07	0.313 b	0 e	2.32 bc
Mean	0.02	0.103	3931	4.51
*p*-Value	-	<0.001	<0.001	0.027
SEM	-	0.158	236.7	1.050
LSD	-	0.048	718	3.184
CV%	-	26.5	10.4	40.3

Values in a column followed by the same letter are not significantly different (*p* = 0.05).

**Table 4:** Content of macro-elements of sweet potato cultivars with varying flesh colors.

Cultivar	K	Cl	*p*	Na	Mg	Ca
	mg 100g^−1^ DW	mg 100g^−1^ DW	mg 100g^−1^ DW	100mg g^−1^ DW	mg 100g^−1^ DW	mg 100g^−1^ DW
Beauregard	1536 a	678.5	153.1 bc	41.4 d	71.7 d	54.8 bc
Bophelo	1284 b	339.8	167.3 b	141.4 a	103.9 b	72.3 ab
Impilo	1494 ab	400.7	198.4 a	97.9 bc	136.2 a	82.5 a
W-119	1004 c	343.0	152.7 bc	121.6 ab	97.9 bc	90.6 a
199062.1	1507 a	407.0	147.1 c	40.8 d	74.6 cd	36.4 c
Monate	1401 ab	366.3	152.3 bc	91.2 bc	76.3 cd	36.9 c
Ndou	1450 ab	388.3	157.8 bc	69.2 cd	87.4 bcd	53.4 bc
Mean	1383	374.8	161.2	86.22	92.58	61.00
*p*-Value	0.001	0.065	0.001	<0.001	<0.001	<0.001
SEM	69.4	16.39	6.50	10.13	7.77	7.38
LSD	210.6	NS	19.71	30.71	23.56	22.38
CV%	8.7	7.6	7.0	20.3	14.5	21.0

Values in a column followed by the same letter are not significantly different (*p* = 0.05).

**Table 5:** Micro-elements contained by sweet potato cultivars with varying flesh colors.

Cultivar	Zn	Mn	Fe	Cu
	mg 100g^−1^ DW	mg 100g^−1^ DW	mg 100g^−1^ DW	mg 100g^−1^ DW
Beauregard	0.577	0.450 c	1.590 bc	0.5767 a
Bophelo	0.663	1.357 a	1.720 ab	0.4500 bc
Impilo	0.730	0.800 bc	1.920 a	0.4367 bc
W-119	0.690	0.873 b	1.727 ab	0.3500 d
199062.1	0.520	0.720 bc	1.400 cd	0.3800 cd
Monate	0.473	0.617 bc	1.280 d	0.3867 cd
Ndou	0.653	0.550 bc	1.587 bc	0.5100 ab
Mean	0.6152	0.767	1.603	0.4414
*p*-Value	0.654	0.007	<0.001	<0.001
SEM	0.075	0.136	0.067	0.0245
LSD	ns	0.411	0.205	0.0743
CV%	21.0	30.6	7.3	9.6

Values in a column followed by the same letter are not significantly different (*p* = 0.05).

**Table 6:** Pearson correlation coefficients of yield versus leaf chlorophyll content index (CCI), glutathione reductase (GR), ascorbate peroxidase (APX), superoxide dismutase (SOD), nitrate reductase (NR), proline content, and ^13^C discrimination (∆^13^C).

			Parameter				
	CCI	GR	APX	SOD	NR	Proline	∆^13^C
Yield	0.521 *	−0.563 *	−0.611 *	−0.508 *	0.764 *	−0.757 *	0.681 *

* Significant at *p* < 0.05.

**Table 7:** Methods used for composite analysis [61].

Analysis	Method	Instrumentation
Moisture	AOAC method 950.46	
Ash	AOAC method 923.03	
Protein	AOAC method 992.23	LECO nitrogen analyzer
Sugars	AOAC method 982.14 (amended)	GC-FID
Starch	AOAC method 996.11	HPLC
Fat	AOAC method 996.06	GC-FID
Total Dietary Fiber	AOAC method 991.43	Enzymatic/gravimetric
Carbohydrates	By calculation (Carbohydrates = 100 − moisture − ash − protein − total fat)	
Energy	By calculation	
	(Energy (in Kcal) = 4 × (proteins and carbohydrates mass in grams) + 9 × mass of fat in grams)(convert to kJ: 1 Kcal = 4.184 kJ)	

AOAC = Association of Official Analytical Chemists.

## Data Availability

Not applicable.
